# Penicillin allergy evaluation: experience from Kuwait

**DOI:** 10.1186/2045-7022-4-S3-P103

**Published:** 2014-07-18

**Authors:** Mona Al-Ahmad, Tito Rodriguez Bouza

**Affiliations:** 1 Allergy Center, Kuwait; 2 Al-Rashed Allergy Center, Allergy Department, Kuwait

## Background

Hypersensitivity to penicillin has been studied worldwide, but data regarding patterns of sensitization in Arabian Gulf countries are scarce.

## Objectives

To describe the patterns of penicillin hypersensitivity during a 6-years study in Kuwait in terms of demographics, type of the culprit drug, in-vivo and in-vitro allergy testing.

## Methods

124 patients referred to the drug allergy clinic for penicillin allergy were fully evaluated by skin prick and intradermal testing. Drug provocation test was done on patients with negative results.

## Results

Total of 124 patients were evaluated for penicillin allergy. Mean age was 37.8 (SD 12.7) years, range from 8 to 74 years. 39 (31.5%) male and 85 (68.5%) female patients were included. Diagnosis of penicillin allergy was confirmed in 46 patients (37.1%). Among the 44 confirmed allergic patients by skin evaluation we had 15 (34.1%) positive skin prick test, and 29 (65.9%) positive intradermal testing. Among patients with positive skin testing, 47.7% were positive to PPL, 20.4% to MDM, 50.0% to PG and 40.9% to ampicillin; 13.6% of patients were positive to amoxicillin by skin prick test. One patient had positive RAST and one had a positive challenge test.

## Conclusion

Penicillin allergy is a common problem with an incidence of about one third in our cohort.

